# N-Acetylcysteine Rescues Hippocampal Oxidative Stress-Induced Neuronal Injury *via* Suppression of p38/JNK Signaling in Depressed Rats

**DOI:** 10.3389/fncel.2020.554613

**Published:** 2020-11-11

**Authors:** Cuiqin Fan, Yifei Long, Liyan Wang, Xiaohang Liu, Zhicheng Liu, Tian Lan, Ye Li, Shu Yan Yu

**Affiliations:** ^1^Department of Physiology, School of Basic Medical Sciences, Cheeloo College of Medicine, Shandong University, Jinan, China; ^2^Morphological Experimental Center, School of Basic Medical Sciences, Cheeloo College of Medicine, Shandong University, Jinan, China; ^3^Shandong Provincial Key Laboratory of Mental Disorders, School of Basic Medical Sciences, Cheeloo College of Medicine, Shandong University, Jinan, China

**Keywords:** oxidative stress, neuroinflammation, apoptosis, N-acetylcysteine, depression

## Abstract

Progression of neuronal deterioration within specific brain regions is considered as one of the principal bases for the development of major depressive disorders. Therefore, protects and promotes the maintaining of normal structure and function of neurons might be a potential therapeutic strategy in the treatment of depression. Here, we report that the antioxidant, N-acetylcysteine (NAC), inhibited neuronal injury through its capacity to reduce oxidative stress and exerted antidepressant effects. Specifically, we show that antioxidant enzyme activity was significantly decreased in the hippocampal CA1 region of depressive rats, while treatment with NAC (300 mg/kg, i.p.) produced neuroprotective effects against mitochondrial oxidative stress injuries and oxidative DNA damage in CA1 neurons of these rats. Moreover, NAC treatment alleviated neuronal injury resulting from neuroinflammation and apoptosis in depressed rats, effects that were associated with reductions in dendritic spine atrophy, and synapse deficits. These effects appear to involve a down-regulation of p38 mitogen-activated protein kinase (MAPK)-JNK signaling along with an up-regulation of ERK signaling within the hippocampal CA1 region. Moreover, this NAC treatment significantly ameliorated depression-like behaviors as indicated by performance in the sucrose preference and forced swim tests (FST). Taken together, these results reveal the potential involvement of oxidative stress in the generation of depression. And, the antidepressant-like effects exerted by NAC may involve reductions in this oxidative stress that can result in neuronal deterioration. Such neuroprotective effects of NAC may indicate a potential therapeutic strategy for the treatment of stress-related depression.

## Introduction

Major depression disorder (MDD) is considered a prevalent psychiatric condition usually resulting from stressful stimuli which then produce neuronal abnormalities and cell death within specific brain regions (Stockmeier et al., 2004; Oh et al., 2012). However, the underlying mechanisms of these pathophysiological effects, and thus the potential for corresponding therapeutic measures, are not fully understood. Previous evidence has indicated that reactive oxygen species (ROS) and reactive nitrogen species (RNS), which are the upstream modulators of both inflammation and apoptosis (Maiuri et al., 2007), represent two critical neural processes believed to be involved in the progression of neuronal injury and the consequent depression-like behaviors (López-López et al., 2016; Fernandes and Gupta, 2019). Interestingly, continuous exposure to stressful stimuli results in the generation of ROS and RNS, the main endogenously reactive oxidizing molecules within cells (Nissanka and Moraes, 2018). And, antioxidant treatment inhibits ROS-induced biochemical and anatomical changes in the ventral tegmental area (VTA), one of the major regions involved in the generation of MDD (Ibi et al., 2017), while overexpression of superoxide dismutase (SOD) has been reported to protect against glucocorticoid-induced depression-like behavioral phenotypes (Uchihara et al., 2016). However, in stress-induced animal models of depression, the related issue of whether therapeutic strategies targeting a disruption in the oxidative stress system can be used as a potential treatment for depression is also currently unknown. Therefore, identification of the effects and mechanisms underlying the oxidative stress as associated with the progression of depression represents critical information required for the development of more effective treatments for this disorder.

Recently, results from clinical studies have indicated that inflammation and the consequent neuronal loss are closely associated with the extent of depression severity, effects which were corroborated from findings showing increased levels of pro-inflammatory cytokines in the peripheral blood circulation and brain regions associated with depression (Dowlati et al., 2010; Maes et al., 2012; Alcocer-Gómez et al., 2015). Similarly, previous results from our laboratory have also revealed that some critical pro-inflammatory cytokines, which respond to environmental stress factors, were significantly elevated in animal models of depression and, were responsible for the neuropathological processes involved with the development of depression-like behavioral phenotypes (Fan et al., 2018, 2019). Related to this issue are the findings that elevated levels of ROS can activate the JNK mitogen-activated protein kinase (MAPK) protein and subsequently lead to the activation of an apoptotic cascade (Katagiri et al., 2010). The findings from our laboratory that inhibition of Cyclooxygenase-2 (COX-2)-mediated inflammation could suppress p38 MAPK activation in an animal model of depression (Song et al., 2018), suggest that, not only may oxidative stress promote neuronal injury to result in the development of depression, but that MAPK signaling may provide a bridge between stress stimuli and neuronal damage, thus revealing a potential pathophysiological mechanism for depression.

The antioxidant, N-acetylcysteine (NAC), has been reported to exert neuroprotective effects in a multitude of psychiatric and neurological disorders (Berk et al., 2013; Bavarsad Shahripour et al., 2014; Slattery et al., 2015). Our previous results also demonstrated that NAC could attenuate inflammatory and apoptotic factors in the dentate gyrus (DG) hippocampus, as well as ameliorates depressive behaviors in rats (Song et al., 2019). However, the underlying neural mechanisms of its possible antidepressant effects are not entirely clear. In this study, we investigated the involvement of oxidative stress in the pathological process of depression as assessed in the chronic unpredictable mild stress (CUMS)-induced animal model of depression, and investigated the mechanisms underlying the neuroprotective effects of the antioxidant, NAC, in the treatment of this condition. We found that oxidative stress was highly activated in the hippocampal CA1 region, one of the key brain regions associated with MDD (Gulyaeva, 2019), and was correlated with depression-like behaviors in these CUMS rats, while NAC greatly promoting microglial anti-inflammatory M2 polarization and ameliorated the morphological changes in nuclei of apoptotic neurons, as well as the display of depression-like behaviors. We further found that NAC could ameliorate the organelle damage, for example, the mitochondria and DNA oxidative damage induced by CUMS exposure. Moreover, pharmacological inhibition of p38 MAPK with SB203580 in these depressed rats suggested that these neuroprotective and antidepressant-like effects of NAC appear to be mediated through suppression of the p38/MAPK-JNK pathway. Taken together, these findings suggest that antioxidants may serve as a potential therapeutic strategy in the treatment of stress-induced depressive disorders.

## Materials and Methods

### Animals

Male Wistar rats (180–200 g body weight) were obtained from the Shandong University Animal Center. Animal experiments were approved by the Ethics Committee of the Animal Experiment Center of Shandong University and were performed according to the National Institutes of Health guidelines for the care and use of laboratory animals. Rats were housed in a temperature and humidity-controlled environment (12 h light/dark cycle) with free access to a standard rat chow diet.

### Depression Model

Depression was induced in this animal model using CUMS exposure as previously described, with minor modifications (Mao et al., 2009). Briefly, the stress regime consisted of 24 h food deprivation followed by 24 h water deprivation, day and night inversion, physical restraint (2 h), cage shaking (2 h), cold swimming (5 min, 4°C), and tail clamping (24 h). Each rat was subjected to one of these stressors in a random order daily for 5 weeks.

### Drug Treatment

The antioxidant, NAC, was purchased from Beyotime Biotechnology (Shanghai, China) and dissolved in normal saline at a concentration of 100 mg/ml. SB203580, the antagonist of p38 MAPK was purchased from Sigma-Aldrich (St. Louis, MO, USA) and dissolved in DMSO (0.1%) at a concentration of 0.25 μg/μl. NAC was administered through an intraperitoneal injection at 60 min before the daily CUMS procedure. The dose and route of NAC administration were based upon previous results demonstrating effective anti-oxidative effects with this protocol (West et al., 2007; Song et al., 2019). Rats were randomly allocated to the following groups (*N* = 18/group): Control (non-stressed), CUMS, CUMS pretreated with NAC (300 mg/kg; NAC + CUMS), Control pretreated with NAC, CUMS pretreated with SB203580 (10 μg/kg; SB + CUMS) and CUMS pretreated with DMSO (0.1%; DMSO + CUMS). SB203580 was administered intracerebroventricularly (i.c.v.) at 60 min before CUMS procedure. A diagram of the experimental design is presented in Supplementary Figure 1.

### Behavioral Tests

#### Open Field Test (OPT)

The open-field test was used to test the spontaneous exploratory activity as described previously (Walsh and Cummins, 1976). Animals were individually placed in the center of an arena (100 × 100 × 40 cm) and were allowed to move freely throughout the arena for a 5-min session. The number of locomotors (segments crossed with the four limbs) and exploratory activities (time stayed in the center zone) were recorded by the observer blind as to the treatment group.

#### Sucrose Preference Test (SPT)

The SPT was administered after the 5 weeks of CUMS as described previously with minor modifications (Mao et al., 2009). Each rat was placed in a cage containing two bottles of 1% sucrose solution for 24 h, then one bottle was replaced with tap water for the following 24 h period. After this adaptation period, food and water were deprived for 24 h followed by the preference test. During the 3 h period of the preference test, each rat was permitted free access to bottles containing either 100 ml of sucrose solution or 100 ml tap water. The sucrose preference was defined as: sucrose consumption/ [water consumption + sucrose consumption] × 100%.

#### Forced Swim Test (FST)

One day after the SPT, the FST was used to assess behavioral despair as reported previously (Duman et al., 2007). Each rat was first placed in a water-filled plastic cylinder (height: 80 cm, diameter: 50 cm, water temperature: 25°C) for 15 min of swimming training. At 24 h after this training session, rats were individually placed in the same cylinder for a 5 min test session. The immobility time (floating, with only movement necessary to maintain their head above the water) of each rat was recorded by an experimenter blind as to the treatment group.

#### Confocal Immunofluorescence Assay

After these behavioral tests, the rats were anesthetized with sodium pentobarbital (40 mg/kg, i.p.) and then perfused with 4% paraformaldehyde (PFA). The rats were then decapitated and the brain isolated for fixing in PFA overnight at 4°C. After graded dehydration, embedded hippocampal brain samples were cut into serial coronal frozen slices (30 μm) and incubated with diluted primary antibodies including polyclonal anti-8-OHdG (1:400, Abcam, Cambridge, UK), anti-ionized calcium-binding adaptor molecule-1 (Iba-1; 1:500, WAKO, Japan) and rabbit anti-glial fibrillary acidic protein (GFAP; 1:100, Proteintech, Waltham, MA, USA) followed by the fluorescent-conjugated secondary antibody (1:200, Sigma-Aldrich). MitoSOX Red fluorescent dye (10 μM, 15 min) was used to examine mitochondrial ROS levels as performed at room temperature. Slices were then washed three times with PBS and incubated with 4′,6-diamidino-2-phenylindole dihydrochloride (DAPI; Thermo Fisher Scientific, Waltham, MA, USA) for 5 min. Images were captured with the use of an LSM780 laser scanning confocal microscope (ZEISS, Germany) System. At least eight presented images from each rat within the four groups (*N* = 6/group) were obtained for analysis using Image-Pro Plus 6.0 software.

### Golgi Staining

Golgi staining was conducted using the FD Rapid GolgiStain™ Kit (PK401, FD Neuro-Technologies, USA) as based on the manufacturers’ instructions. Rats were anesthetized, brains removed, and immersed in the soak solution (A/*B* = 1:1, 15 ml/rat) in the dark at room temperature for 2 weeks. Brains were cut into coronal slices (100 μm), cleaned in xylene, and cover-slipped with Rhamsan gum for light microscopic observation. At least six apical dendritic segments per pyramidal neuron were captured, and six to eight pyramidal neurons were chosen from each rat for morphological analysis. Spine densities were measured with the use of Image-Pro plus software.

### Transmission Electron Microscopy

Transmission electron microscopy (Philips Tecnai 20, Holland) was used to observe the ultrastructure of CA1 neurons. Rats were anesthetized and the CA1 region (1 mm × 1 mm × 1 mm) was dissected and placed in 2.5% glutaraldehyde for 4 h. After dehydration with ethanol gradients, samples were immersed in propylene oxide overnight and embedded with resin. Samples were cut into slices (70 nm thick) and stained with 4% uranyl acetate followed by 0.5% lead citrate on the copper grids. A minimum of 30 micrographs was obtained from each rat for measurements using ImageJ analysis software.

### Reverse Transcription PCR

Total RNA was extracted from CA1 tissue using the Trizol Reagent (Invitrogen, USA) and reverse-transcribed into cDNA with the use of the primeScript RT reagent kit (TaKaRa, Otsu, Japan). The cDNA was amplified by PCR using specific primers (Supplementary Table 1). PCR products were assessed by electrophoresis on a 3% agarose gel and were detected with the use of the Gel Image Analysis System (Bio-rad, USA). Levels of targeted mRNA were normalized to GAPDH.

### Western Blot Analysis

CA1 tissues were homogenized in RIPA lysis buffer containing a protease and phosphatase inhibitor. Equal amounts of protein (30 μg) were subjected to SDS-PAGE gels and then transferred to the PVDF membranes. The membrane was blocked using 5% nonfat milk and incubated with the primary antibodies, anti-phospho-ERK (1:1,000, Cell Signaling Technology, Danvers, MA, USA), anti-phospho-p38 MAPK (1:1,000, Cell Signaling Technology, Danvers, MA, USA), anti-phospho-JNK (1:1,000, AbCam, USA) or anti-β-actin (1:8,000, Santa Cruz Biotechnology, Dallas, TX, USA). After incubation with the HRP-conjugated secondary antibody, the image was acquired with the use of the ChemiDocTM Imager from Bio-Rad. Protein bands were quantified with the use of ImageJ software and were normalized to β-actin.

### Oxidative Stress Measurements

Enzyme activity was detected using the glutathione peroxidase (GSH-Px) activity assay Kit (No. A005) and the SOD activity assay Kit (No. A001-3) according to the manufacturers’ instructions. The content of malondialdehyde (MDA; No. A003-1) and nitric oxide (NO; No. A013-2) were detected with the use of the assay kits based on the manufacturers’ guidelines. All assay kits were purchased from Jiancheng Incorporation (Nanjing, China).

### Statistical Analysis

Data were presented as the mean ± SEM. Statistical analyses were performed using either two-tailed Student’s *t*-tests or two-way analysis of variance (ANOVA) followed by Tukey’s multiple-comparison test when appropriate. A *P*-value < 0.05 was required for results to be considered as statistically significant. SPSS version 17.0 was used for these analyses.

## Results

### NAC Suppressed Oxidative Stress Activity and Depression-Like Behaviors in Rats

**Figure 1:**
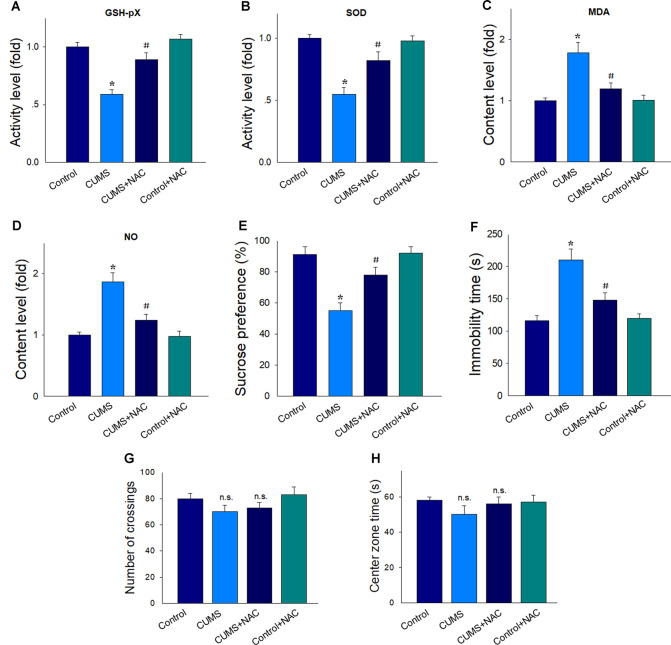
NAC suppresses oxidative stress in the hippocampal CA1 region of chronic unpredictable mild stress (CUMS) rats and rescued depression-like behaviors. In CUMS rats NAC. **(A)** Elevated the decreased activity of the antioxidant enzyme GSH-pX. **(B)** Elevated the decreased activity of the antioxidant enzyme superoxide dismutase (SOD). **(C)** Decreased the contents of malondialdehyde (MDA). **(D)** Decreased the contents of nitric oxide (NO). **(E)** Prevented the decreased consumption of sucrose solution. **(F)** Reversed the increases in immobility times in the forced swim test (FST). **(G)** Have no effects on the locomotion in the open field test (OPT). **(H)** Have no effects on the exploratory activities in rats. For behavioral assessments, *N* = 18 per group. In other groups, *N* = 6. *P < 0.05, compared to the Control group; ^#^*P* < 0.05, compared to CUMS group; n.s., no significance; NAC, N-acetylcysteine.

As shown in Figure 1, similar to our recent studies (Song et al., 2019), the activity of antioxidant enzymes SOD (Figure 1A) and GSH-pX (Figure 1B) were significantly reduced after 5-weeks of CUMS exposure compared with that in non-stressed rats (*P* < 0.05). However, treatment with the antioxidant, NAC, significantly increased antioxidant enzyme activities in CUMS rats (*P* < 0.05). Accordingly, levels of the oxidative stress products, MDA and NO within the CA1 region were significantly elevated after CUMS exposure (*P* < 0.05) and significantly suppressed by NAC pretreatment (*P* < 0.05; Figures 1C,D). CUMS exposure induced depression in rats as observed by the display of anhedonia in the SPT (*P* < 0.05; Figure 1E), as well as by the increases in immobility times in the FST (*P* < 0.05; Figure 1F). More importantly, NAC treatment significantly ameliorated these depression-like behavioral phenotypes (*P* < 0.05). Also, NAC treatment has no significant effects on the locomotor activity of rats (*P* > 0.05; Figure 1G), and has no effects on the exploratory activities which usually presented the anxiety in rats (*P* > 0.05; Figure 1H). There were no significant differences between the NAC treated non-stressed control group and the non-stressed control group (*P* > 0.05), which suggests that this dose of NAC produced no antioxidant defenses in control conditions. Therefore, in the following experiments, the use of the NAC treated non-stressed control group was discontinued to decrease the number of animals used.

### NAC Attenuates Mitochondria and DNA Oxidative Damage in Depressed Rats

**Figure 2:**
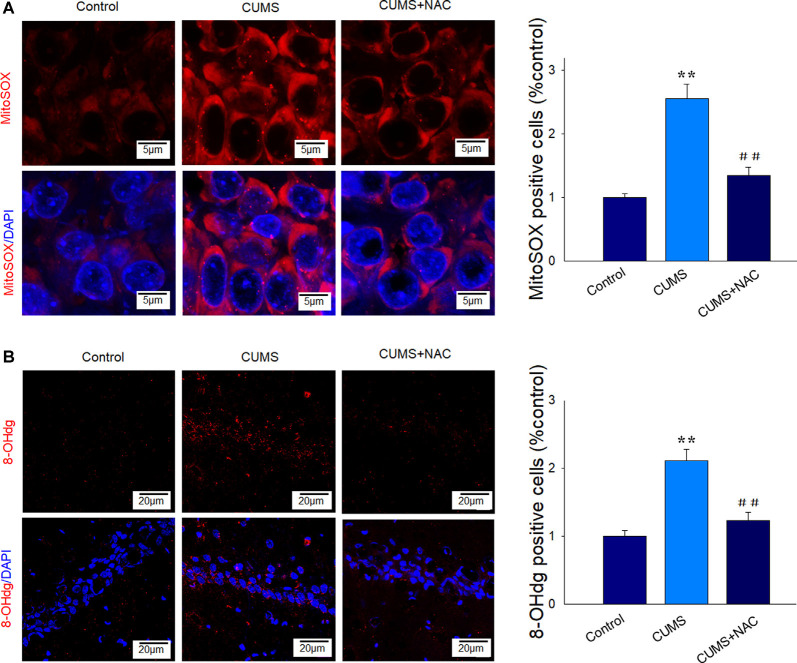
NAC suppresses mitochondria and DNA oxidative damage in the hippocampal CA1 region of CUMS rats. **(A)** Representative images of Mito-SOX staining (red). Nuclei (blue) are stained with 4′,6-diamidino-2-phenylindole dihydrochloride (DAPI). The scale bar is 5 μm. **(B)** Representative images of 8-OHdG staining (red). Nuclei (blue) are stained with DAPI. The scale bar is 20 μm. *N* = 6 per group. **P < 0.01, compared to Control group; ^##^*P* < 0.01, compared to CUMS group; NAC, N-acetylcysteine.

Levels of MitoSox were significantly increased within the CA1 region of depressed rats compared to the non-stressed controls, which imply that this CUMS exposure resulted in higher levels of mitochondrial superoxides (*P* < 0.01). These responses were effectively alleviated in depressed rats receiving NAC pretreatment (*P* < 0.01; Figure 2A). We then examined the degree of oxidative DNA damage in depressed rats as based on 8-oHdG levels, a marker of oxidative base damage. We found that the expression of 8-oHdG was significantly increased in depressed rats (*P* < 0.01), effects that were reversed by NAC treatment (*P* < 0.01; Figure 2B). These results suggest that NAC effectively reduced oxidative stress damage within the hippocampal CA1 region of CUMS rats.

### NAC Attenuates Neuro-inflammatory Responses in Depressed Rats

**Figure 3:**
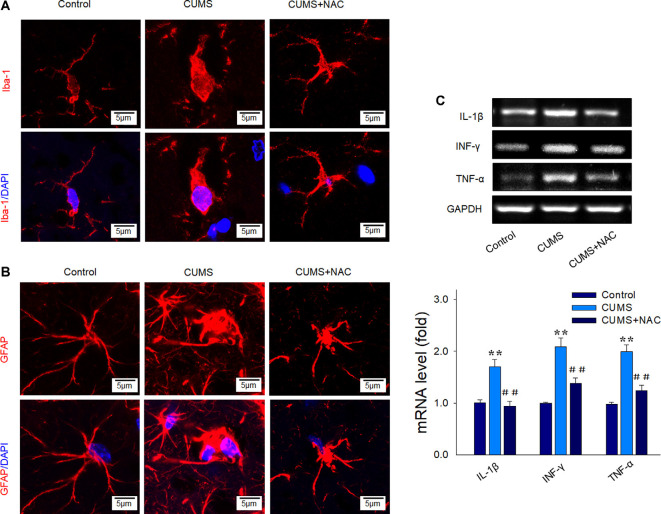
NAC suppresses glial activation and inflammatory cytokine expressions in the hippocampal CA1 region of CUMS rats. **(A)** Immunofluorescent signals of Iba1 positive microglial cells within the CA1 region. Microglial cells retracted their processes and assumed a rounded morphology after CUMS exposure, effects that were attenuated by NAC pretreatment. Nuclei (blue) are stained with DAPI. The scale bar is 5 μm. **(B)** Immunofluorescent signals of glial fibrillary acidic protein (GFAP) positive astrocytes within the CA1 region. NAC pretreatment attenuated the activation of astrocytes. Nuclei (blue) are stained with DAPI. The scale bar is 5 μm. **(C)** RT-PCR assays of mRNA expression levels of IL1β, TNF-α, and IFN-γ within each group. *N* = 6 per group. **P < 0.01, compared to Control group; ^##^*P* < 0.01, compared to CUMS group; NAC, N-acetylcysteine.

Results from our immunofluorescent assay revealed that chronic stress significantly activated microglia within the CA1 region, as indicated by increases in cell soma and retraction of ramified processes (Figure 3A). Such CUMS-induced morphological changes were also observed for astrogliosis (Figure 3B). Importantly, not only were these glial activations significantly alleviated by NAC pretreatment, but the elevated mRNA levels of the pro-inflammatory cytokines IL-1β, IFN-γ, and TNF-α resulting from CUMS were also reversed by NAC treatment (*P* < 0.01; Figure 3C), similar to that observed in DG regions in our previous study (Song et al., 2019). These results suggest that increased levels of oxidative stress could generate neuro-inflammatory responses, which may, in turn, induce the depression-like behaviors observed in these rats.

### NAC Rescues Neuronal Synaptic Deficits and Spine Density Loss in Depressed Rats

**Figure 4:**
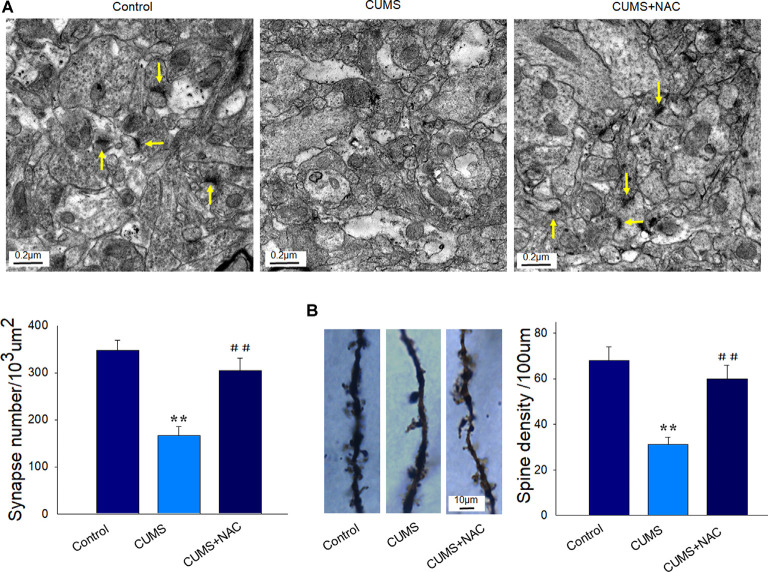
NAC rescues neuronal synaptic deficits and spine loss in the hippocampal CA1 region of CUMS rats.** (A)** Representative electron micrograph of CA1 neurons in rats. Arrows indicate spine synapses. The scale bar is 0.2 μm. **(B)** Golgi staining images of spine densities of dendrites within CA1 regions. The scale bar is 10 μm. *N* = 6 per group. **P < 0.01, compared to Control group; ^##^*P* < 0.01, compared to CUMS group; NAC, N-acetylcysteine.

Results obtained from TEM analysis showed that the number of synapses within CA1 neurons of depressed rats was significantly reduced as compared to the non-stressed rats (*P* < 0.01). However, these deficiencies in synapse number by CUMS exposure were significantly improved by NAC treatment (*P* < 0.01; Figure 4A). Moreover, our results from Golgi staining demonstrated that dendritic spine loss in CA1 neurons induced by CUMS exposure was also significantly reversed by NAC treatment (*P* < 0.01; Figure 4B). The restore of synaptic and spine deficits is similar to the protective effects of NAC in DG regions (Song et al., 2019). These results reveal that the oxidative stress induced by CUMS produced a deterioration of the neuronal structure, while NAC treatment provided neuroprotective effects against these changes.

### NAC Attenuates Neural Apoptosis in Depressed Rats

**Figure 5:**
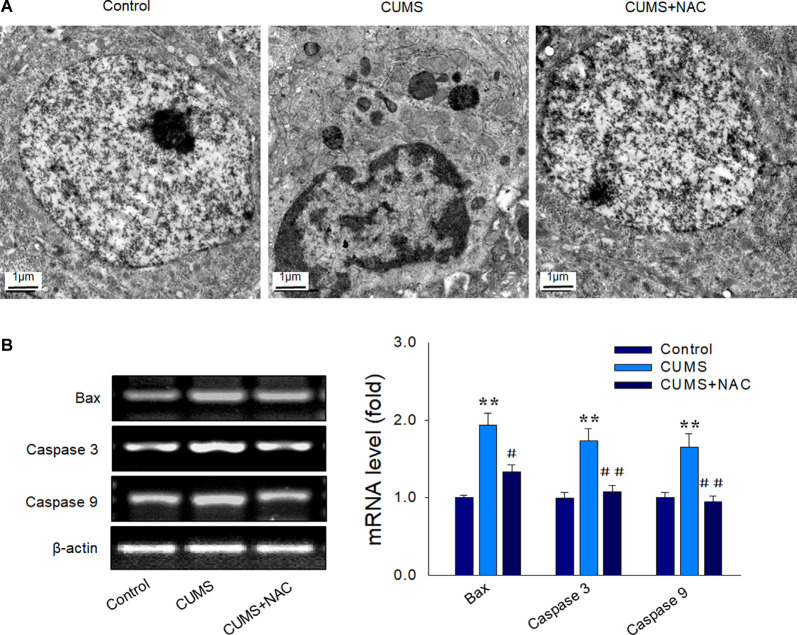
NAC suppresses neural apoptosis in the hippocampal CA1 region induced by CUMS exposure. **(A)** Representative electron micrograph of CAI neuronal ultrastructure revealing nuclear chromatin aggregation, condensation, and margination. The scale bar is 1 μm. **(B)** NAC reduced mRNA expression levels of Bax, cleaved caspase 3, and caspase 9 within CA1 regions of CUMS rats. *N* = 6 per group. **P < 0.01, compared to Control group; ^#^*P* < 0.05, ^##^*P* < 0.01, compared to CUMS group; NAC, N-acetylcysteine.

Results from our TEM analysis also revealed that CUMS-exposure produced clear morphological changes in nuclei, including nuclear chromatin margination, aggregation, and condensation, while NAC pretreatment significantly mitigated these characteristics of apoptosis in CA1 neurons (Figure 5A). Also, increased mRNA levels of the pro-apoptotic factors Bax, caspase 3, and caspase 9 induced by CUMS exposure were all significantly alleviated by NAC treatment (*P* < 0.01; Figure 5B). These results suggest that the initial increase in oxidative damage within the CA1 region, resulting from CUMS-exposure may be one of the triggers of inflammation and apoptosis, subsequently leading to neuronal injury.

### NAC Rescues Neural Injury Through Suppression of the p38/JNK MAPK Pathway

**Figure 6:**
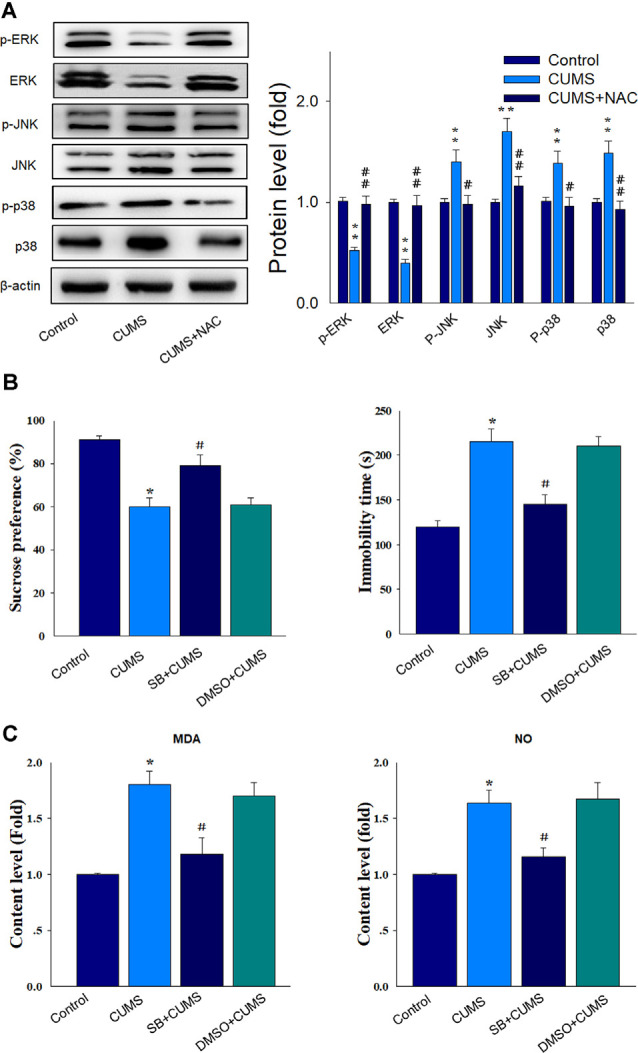
NAC rescues neuronal injury and depression-like behaviors *via* suppression of the p38/JNK mitogen-activated protein kinase (MAPK) pathway. **(A)** Western blot analysis of the phosphorylated and total ERK, p38, and JNK protein expressions within each group. **(B)** Pretreatment of SB203580 ameliorated the depression-like behaviors in CUMS rats. **(C)** The pretreatment of SB203580 reduced the contents of MDA and NO in CUMS rats. *N* = 6. *P < 0.05, **P < 0.01, compared to Control group; ^#^*P* < 0.05, ^##^*P* < 0.01, compared to CUMS group; NAC, N-acetylcysteine.

Results from the western blot assay showed that the phosphorylated and total protein levels of ERK were elevated by NAC treatment (*P* < 0.01), while the phosphorylated and total protein levels of p38 and JNK were significantly attenuated by NAC treatment in CUMS rats (*P* < 0.01; Figure 6A). Then we used SB203580, the antagonist of p38 MAPK to verify whether the p38 MAPK pathway may contribute to the activation of oxidative stress and involved in the protective effects of NAC in depressive rats. The behavioral results showed that SB203580 significantly reversed the depression-like symptoms caused by CUMS exposure (*P* < 0.05; Figure 6B). Moreover, levels of the oxidative stress products, MDA and NO, which elevated by CUMS exposure (*P* < 0.05) were also significantly suppressed by SB203580 pretreatment (*P* < 0.05; Figure 6C). Such findings suggest that activation of the p38MAPK and JNK pathways may be involved in promoting the effects of oxidative stress upon inflammatory responses. When combining all results of this report, we proposed that NAC, in part, can exert anti-depressant-like effects through its capacity to initially suppress oxidative stress. This suppression of oxidative stress can then reduce the inflammatory and apoptotic responses associated with stress through suppression of the p38/MAPK-NK signaling pathway, ultimately alleviating the display of depression-like behaviors in this animal model of depression.

## Discussion

In the present study we investigated the neuroprotective effects of the antioxidant, NAC, and thus revealed some of the underlying mechanisms of its antidepressant-like effects in a rat model of depression. Our previous article (Song et al., 2019) found that NAC could reduce the increased expression of inflammatory cytokines and apoptotic factors in DG regions induced by CUMS exposure. In the present study, to further verify the anti-inflammatory effects of NAC, we examined the activity of glial cells and found that microglia, the most important immune-inflammatory cell in the central nervous system (CNS), showed enlargement of cell body through morphological detection, which indicated it is in a secretory state after 5-weeks chronic stress. The activation of microglia could be suppressed by NAC, which suggested that NAC treatment restored behavioral deficits after CUMS in rats, in part, involved promoting microglial anti-inflammatory M2 polarization in CA1 hippocampus. For neural apoptosis induced by CUMS exposure, we further revealed CUMS caused morphological changes in nuclei, including nuclear chromatin margination, aggregation, and condensation, the characteristic changes which indicated the apoptosis of neurons, while NAC pretreatment significantly mitigated these characteristics of apoptosis in the CA1 region. Moreover, our results demonstrated that NAC treatment significantly suppressed oxidative stress activity in depressed rats. This protective effect was accompanied by an attenuation of mitochondrial and DNA oxidative damage. Besides, CUMS rats treated with NAC also showed amelioration in dendritic spine loss and synaptic deficiencies, along with a reduction in the display of apoptosis in CA1 neurons, effects similar to that found in DG regions in our previous study (Song et al., 2019). The potential involvement of oxidative stress in the generation of depression was corroborated from results showing that NAC pretreatment significantly attenuated the genesis and progress of depression-like behaviors in these CUMS rats. Moreover, these processes appear, in part, to result *via* modulation of the p38/JNK pathway. Therefore, when taken together these findings not only demonstrate the neuroprotective effect of NAC but also reveal some of the mechanisms involved and suggest a potential, new strategy in the treatment of depression.

In this study, we found that the activity of antioxidant enzymes was significantly down-regulated and expression levels of oxidative stress products elevated within hippocampal regions in this rat model of depression. In response to excessive production of oxidative stress and peroxidation, mitochondrial oxidative stress was then elevated, as indicated by increased expressions of Mito-SOX. Besides, this CUMS-induced enhancement of oxidative stress also resulted in oxidative DNA damage, as indicated by increased levels of the DNA damage marker, 8-OHdG. However, pretreatment with the antioxidant, NAC, effectively prevented the up-regulation of mitochondrial stress and DNA damage resulting from this CUMS exposure. It has been reported that mitochondrial dysfunction may play a considerable role in the development of depression (Bansal and Kuhad, 2016). Under stress condition, mitochondria represent the major site for ROS generation, typically characterized as toxic molecules which activate neuronal inflammatory responses (Forrester et al., 2018). There is increasing evidence that neuroinflammatory responses denote a critical risk factor in the development of depression (Haapakoski et al., 2015; Rosenblat et al., 2016; Park et al., 2018). To further substantiate a role for NAC in these neuroprotective effects as achieved through inhibiting oxidative stress and the consequent neuronal inflammation and apoptosis, we tested the effects of this antioxidant in this CUMS model. As NAC has been found to reduce the levels of oxidative stress markers, as well as decrease the reperfusion-induced burst production of ROS and the consequent inflammation (Wang et al., 2017), we deduced that it may serve as a worthwhile agent for use in examining some of the potential neuropathological processes involved in depression. As an initial step toward this goal, we first established that our CUMS rat model produced neuroinflammation and apoptosis within the hippocampal CA1 region in these rats. We then assessed the effects of NAC pretreatment in these CUMS rats. Significant protective effects of this NAC treatment were observed as indicated by decreases in glial activation and the pro-inflammatory markers IL-1β, IFN-γ, and TNF-α, as well as by inhibition in neuronal apoptosis in the hippocampal CA1 area, all of which were aggravated by CUMS exposure. We also found that NAC markedly ameliorated dendritic spine loss and synaptic deficits within CA1 neurons in depressed rats, suggesting that the oxidative stress observed in these CUMS rats might then lead to neuronal deterioration. Together with what we found in DG regions in our previous study (Song et al., 2019), these neuroprotective effects of NAC suggest critical underlying mechanisms of its antidepressant-like effects in rats. That these findings have clinical relevance are revealed from the results of clinical studies showing progressive atrophy and reduction in hippocampal volume in patients with MDD (Koolschijn et al., 2009; Savitz et al., 2015). Moreover, the anhedonia and behavioral despair observed in these CUMS rats are similar to that observed in major depression patients (Wang et al., 2018; Gupta and Fernandes, 2019; Zhang et al., 2019).

An additional mechanistic component identified in this study was the observation of significant increases in phosphorylation levels of MAPK pathway proteins such as c-Jun, N-terminal kinase (JNK), and p38 in these CUMS rats. In addition to the enhanced activity of the p38/JNK pathway, we also found that phosphorylation levels of ERK were significantly decreased by CUMS exposure. All of these effects resulting from CUMS were reversed by NAC treatment. Moreover, to determine whether the p38 MAPK pathway may contribute to the activation of oxidative stress and whether p38 involved in the protective effects of NAC in depressive rats, we injected SB203580 to block p38 MAPK activity before CUMS exposure, and the behavioral results showed that SB203580 significantly ameliorate the depression-like symptoms and significantly reduced the levels of the oxidative stress products, MDA and NO, which induced by CUMS exposure. These results suggested that p38 MAPK may contribute to the oxidative stress and depression-like behaviors in rats. Taken together, these results indicate that antioxidants may attenuate neuroinflammation and apoptosis within the hippocampus of CUMS rats *via* down-regulating the p38/JNK MAPK pathway, while ERK survival signaling might be one of the main down-stream regulators of NAC in the regulation of hippocampal neuronal injury and display of depression-like behaviors. While results from a previous study have indicated that pro-inflammatory cytokines could activate the p38 MAPK protein leading to decreased synaptic availability of serotonin and depressive-like behavior (Adzic et al., 2018), we found that inhibition of Cyclooxygenase-2 (COX-2)-mediated inflammation could suppress p38 activation in this animal model of depression (Song et al., 2018). It has also been reported that induction of apoptotic cell death is associated with increased levels of ROS (McManus et al., 2014; Morris and Berk, 2015), which subsequently promote the activation of the JNK protein, an effector molecule of the apoptotic cascade (Katagiri et al., 2010). When collating these results, they suggest that NAC, in part, can exert neuroprotective effects through its capacity to initially suppress oxidative stress which then reduces the inflammatory and apoptotic responses through suppression of the p38/MAPK-NK signaling pathway in this animal depression model, ultimately ameliorating the display of depression-like behaviors in these rats. These observations suggest that the over-activation of oxidative stress may function as a key contributor in the promotion of neuroinflammation, which may then result in the neuronal injury and behavioral phenotypes in depression. Thus, the prevention of this initial onset of oxidative stress by NAC may prevent the eventual neural deterioration involved with depression and, in this way, serve as a critical mechanism for the antidepressant-like effects of NAC.

In conclusion, our current study provides strong evidence that the antioxidant, NAC, protects against neuronal injury and depressive behaviors caused by oxidative stress. This effect appears to, at least in part, be exerted using its neuroprotective effects which result in anti-inflammatory and anti-apoptosis effects in depressed rats. These findings suggest that targeting inhibition of oxidative stress might serve as a potential therapeutic strategy for the treatment of depressive disorder.

## Data Availability Statement

The raw data supporting the conclusions of this article will be made available by the authors, without undue reservation.

## Ethics Statement

The animal study was reviewed and approved by the Ethics Committee of the Animal Experiment Center of the Shandong University and were performed according to the International Guiding Principles for Animal Research provided by the International Organizations of Medical Sciences Council.

## Author Contributions

CF and SY contributed to the experimental design and analyses of data. YiL and XL performed the Western blot analysis, qPCR, and ELISA. LW and ZL contributed to the TEM analysis. YeL and TL performed confocal imaging. TL constructed a depression model and behavioral tests. SY wrote the draft. CF and YeL participated in the revision. All authors contributed to the article and approved the submitted version.

## Conflict of Interest

The authors declare that the research was conducted in the absence of any commercial or financial relationships that could be construed as a potential conflict of interest.
