# Supplementary material for: N-Acetylcysteine Rescues Hippocampal Oxidative Stress-Induced Neuronal Injury via Suppression of p38/JNK Signaling in Depressed Rats
Source: Front Cell Neurosci. 2020 Nov 11;14:554613. doi: 10.3389/fncel.2020.554613 (PMC7686549; doi:10.3389/fncel.2020.554613)
Supplement: Supplementary file 1 [file Table_1.DOCX]

**Supplemental Table 1.** PCR primers used in this study

| *Gene* | *Forword (*5’→3’*)* | *Reverse (*5’→3’*)* |
| --- | --- | --- |
| IL-1β | AAG ATG AAG GGC TGC TTC CAA ACC | ATA CTG CCT GCC TGA AGC TCT TGT |
| IFN-γ | ATT CAT GAG CAT CGC CAA GTT C | TGA CAG CTG GTG AAT CAC TCT GAT |
| TNF-α | TGA TCG GTC CCA ACA AGG A | TGC TTG GTG GTT TGC TAC GA |
| Bax | TCT TCA AAC TGC TGG GCC ATT | CTT GTC ACC TGC CTG ACT GCT |
| Caspase3 | GGA GCT TGG AAC GCG AAG AA | ACA CAA GCC CAT TTC AGG GT |
| Caspase9 | CAA GAA GAG CGG TTC CTG GT | CAG AAA CAG CAT TGG CGA CC |
| GAPDH | AGT GCC AGC CTC GTC TCA TA | GGT AAC CAG GCG TCC GAT AC |
