# Supplementary material for: N-Acetylcysteine Rescues Hippocampal Oxidative Stress-Induced Neuronal Injury via Suppression of p38/JNK Signaling in Depressed Rats
Source: Front Cell Neurosci. 2020 Nov 11;14:554613. doi: 10.3389/fncel.2020.554613 (PMC7686549; doi:10.3389/fncel.2020.554613)
Supplement: Supplementary file 2 [file Table_2.DOCX]

**SUPPLEMENTARY FIGURE LEGENDS**

**N-acetylcysteine rescues oxidative stress-induced neural injury in hippocampus via suppression of p38/JNK signaling in depressed rats**

Cuiqin Fan^1^, Yifei Long^1^, Liyan Wang^2^, Xiaohang Liu, Zhicheng Liu, Tian Lan^1^, Ye Li^1^ , Shu Yan Yu^1, 3^﹡

1. Department of Physiology, Shandong University, School of Basic Medical Sciences, 44 Wenhuaxilu Road, Jinan, Shandong Province, 250012, PR China;

2. Morphological experimental center, Shandong University, School of Basic Medical Sciences, 44 Wenhuaxilu Road, Jinan, Shandong Province, 250012, PR China;

3. Shandong Provincial Key Laboratory of Mental Disorders, School of Basic Medical Sciences, 44 Wenhuaxilu Road, Jinan, Shandong Province, 250012, PR China;

﹡ Corresponding author: Shu Yan Yu,

E-mail address: shuyanyu@sdu.edu.cn

Tel: +86-0531-88383902; fax: +86-0531-88382502

**Figure legnds:**

**S Fig.1. Depression animal model experimental design: schematic figure of the treatment protocol of rats.** CUMS, chronic unpredictable mild stress; i.p., intraperitoneal; NAC, N-acetylcysteine; SPT, sucrose preference test; FST, forced swim test.
