# Supplementary figures and images for: N-Acetylcysteine Rescues Hippocampal Oxidative Stress-Induced Neuronal Injury via Suppression of p38/JNK Signaling in Depressed Rats
Source: Front Cell Neurosci. 2020 Nov 11;14:554613. doi: 10.3389/fncel.2020.554613 (PMC7686549; doi:10.3389/fncel.2020.554613)

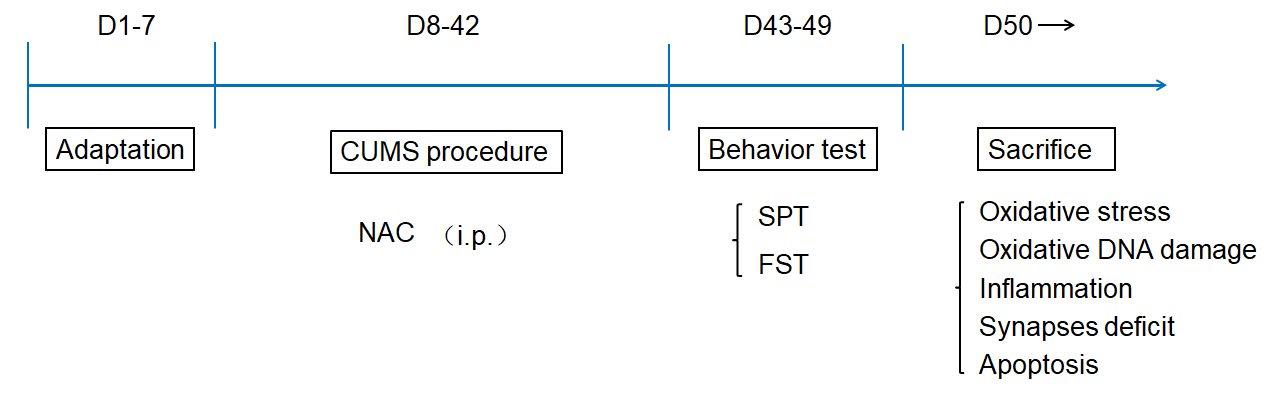

Supplement: Supplementary file 3 [file Image_1.TIF]
